# Characterizing the residual glass in a MgO/Al_2_O_3_/SiO_2_/ZrO_2_/Y_2_O_3_ glass-ceramic

**DOI:** 10.1038/srep34965

**Published:** 2016-10-13

**Authors:** Sabrina Seidel, Christian Patzig, Wolfgang Wisniewski, Antje Gawronski, Yongfeng Hu, Thomas Höche, Christian Rüssel

**Affiliations:** 1 Otto Schott Institut, Jena University, Fraunhoferstraße 6, D-07743 Jena, Germany; 2 Fraunhofer Institute for the Microstructure of Materials and Systems IMWS, Walter-Huelse-Straße 1, 06120 Halle (Saale), Germany; 3 Canadian Light Source Inc., University of Saskatchewan, 44 Innovation Boulevard, Saskatoon, S7N 2V3, Canada

## Abstract

The non-isochemical crystallization of glasses leads to glass-ceramics in which the chemical composition of the amorphous matrix differs from that of the parent glass. It is challenging to solely analyse the properties of these residual glassy phases because they frequently contain finely dispersed crystals. In this study, the composition of the residual glass matrix after the crystallization of a glass with the mol% composition 50.6 SiO_2_ · 20.7 MgO · 20.7 Al_2_O_3_ · 5.6 ZrO_2_ · 2.4 Y_2_O_3_ is analysed by scanning transmission electron microscopy (STEM) including energy dispersive X-ray analysis (EDXS). A batch of the residual glass with the determined composition is subsequently melted and selected properties are analysed. Furthermore, the crystallization behaviour of this residual glass is studied by X-ray diffraction, scanning electron microscopy including electron backscatter diffraction and STEM-EDXS analyses. The residual glass shows sole surface crystallization of indialite and multiple yttrium silicates while bulk nucleation does not occur. This is in contrast to the crystallization behaviour of the parent glass, in which a predominant bulk nucleation of spinel and ZrO_2_ is observed. The crystallization of the residual glass probably leads to different crystalline phases when it is in contact to air, rather than when it is enclosed within the microstructure of the parent glass-ceramics.

Glasses and glass-ceramics in the system MgO/Al_2_O_3_/SiO_2_ (MAS) are suitable for many applications^1^,^2^,^3^,^4^, especially with respect to their good mechanical properties such as hardness, Young’s modulus, strength and fracture toughness^4^,^5^,^6^,^7^,^8^. Many compositions in the MAS system predominantly show surface crystallization, for example that of cordierite^9^,^10^,^11^,^12^. By contrast, adding nucleating agents such as TiO_2_^5^,^13^,^14^,^15^,^16^ or ZrO_2_^4^,^6^,^7^,^8^,^16^,^17^,^18^ may lead to bulk crystallization in MAS glasses. The required concentration of these nucleation agents is typically in the range from 5–8 mol% for stoichiometric cordierite glasses^4^,^6^,^7^,^8^. If the mentioned nucleation agents are added, a first annealing step at temperatures some 10 K above the glass transition temperature *T*_*g*_ typically leads to the precipitation of TiO_2_ or ZrO_2_ nano-crystals^17^. Applying a subsequent thermal treatment leads to the crystallization of a high-quartz solid solution^17^ (high-QSS, also known as β-QSS^4^,^10^,^19^ or μ-cordierite^3^,^7^,^20^), which is stabilized at room temperature due to the incorporation of considerable quantities (ca. 10 mol%) of both MgO and Al_2_O_3_^4^,^5^. Typically, the high-QSS is depleted of MgO and Al_2_O_3_ at higher crystallization temperatures which results in a phase transition to the low-quartz solid solution (low-QSS) during cooling^4^,^5^. Simultaneously, the precipitation of spinel (MgAl_2_O_4_) is observed^4^,^17^,^18^. It is often argued that the phase transition of the high-QSS (coefficient of thermal expansion (CTE) <3.5 · 10^−6^ K^−1^ 21) into the low-QSS during cooling is essential to raise the mechanical strengths of the glass-ceramics up to 475 MPa^4^. These high strengths result from both the volume contraction of 0.8% during the phase transition and the large CTE mismatch between the low-QSS (CTE_(20−300 °C)_ = 13.2 · 10^−6^ K^−1^ 22) and the residual glassy phase^4^,^18^.

It was recently described that adding Y_2_O_3_ in concentrations from 0.5 to 5 mol% to a stoichiometric cordierite glass decelerates the crystallization process^2^,^6^,^7^ and may also prevent the crystallization of the high-/low-QSS^6^,^7^,^22^. This was especially shown in a glass with the mol% composition 50.6 SiO_2_ · 20.7 MgO · 20.7 Al_2_O_3_ · 5.6 ZrO_2_ 2.4 Y_2_O_3_^6^,^7^,^23^. A considerable quantity of residual glass was detected after crystallizing at 950 °C for 5 h, cooling to room temperature (RT) and a second crystallization step at 1060 °C for 1 h^6^,^23^. However, the produced glass-ceramics, containing tetragonal (or cubic) ZrO_2_ (CTE = 10.5 · 10^−6^ K^−1^ 24) and spinel (MgAl_2_O_4_, CTE_(20–800 °C)_ = 8 · 10^−6^ K^−1^ 22), show excellent mechanical properties despite the absence of any QSS^7^. It was argued that the mismatch of the large CTE of both ZrO_2_ and spinel compared to the CTE of the residual glassy phase is most probably sufficient to produce high-strength glass-ceramics^7^. This argumentation followed the assumption that the CTE of the residual glass is close to the CTE of the original glass. Up to now, this assumption has not been supported by experimental results because the residual glass frequently occurs in small pockets in the μm- or even nm-range. This has made it very challenging to experimentally determine its properties so far, unless the crystalline phases were removed, e.g. by chemical etching, which is usually not possible without changing the residual glass itself.

In another study, these glass-ceramics have been intensively investigated using analytical scanning transmission electron microscopy (STEM) and X-ray absorption at near edge spectroscopy (XANES) at the Zr*L*_*2*_-, Y *L*_*2,3*_-, Si *K*- and Al *L*- edges^23^. The composition of the residual glassy phase was determined using STEM in combination with energy-dispersive X-Ray spectroscopy (EDXS), and it was found that a considerable amount of Zr is still present in the residual glass, showing that not all Zr contributed to the crystallization of ZrO_2_ in these glass-ceramics. Additionally, the coordination of the respective elements in the samples was analysed, and the change of the Zr coordination from 6-fold (in the glass) to 8-fold (after the crystallization of ZrO_2_) was reported. However, a direct measurement of the coordination of the remaining Zr in the residual glassy phase was not possible due to the too small areas of residual glass with respect to the lateral resolution of the XANES analyses^23^.

Not only the MAS glass-ceramics analyzed here, but many other examples of (partially) crystallized glass-ceramics contain amorphous phases which have a composition different from that of the parent glass. During the course of such a non-isochemical crystallization, the chemical composition of the glass matrix changes with time, which also leads to a time dependent crystal growth velocity. In some cases, the viscosity of the glass matrix increases until its glass transition temperature *T*_*g*_ is equal to the supplied annealing temperature^18^,^25^,^26^,^27^, which completely freezes the crystallization. This effect has recently been described for several glass systems, e.g. during the crystallization of ZrO_2_ from a MgO/Al_2_O_3_/SiO_2_/ZrO_2_ glass^18^, during the crystallization of BaF_2_ from a Na_2_O/K_2_O/BaF_2_/Al_2_O_3_/SiO_2_ glass^25^, during the crystallization of CaF_2_ from a Na_2_O/K_2_O/CaO/CaF_2_/Al_2_O_3_/SiO_2_ glass^26^ and during the crystallization of ZrTiO_4_ from a Li_2_O/Na_2_O/K_2_O/MgO/BaO/ZnO/Al_2_O_3_/SiO_2_/TiO_2_/ZrO_2_/As_2_O_3_ glass^27^. Sometimes, a comparably large quantity of amorphous matrix remains although its *T*_*g*_ should be far below the supplied crystallization temperature, which can be as high as 1000 to 1100 °C. An experimental access to the properties of the residual glass would be of great interest in all of these systems.

In this paper, the residual glass in glass-ceramics resulting from the crystallization of a glass with the mol% composition 50.6 SiO_2_ · 20.7 MgO · 20.7 Al_2_O_3_ · 5.6 ZrO_2_ · 2.4 Y_2_O_3_ is intensively investigated concerning its thermal properties (CTE, *T*_*g*_), its density, the coordination of Zr and its mechanical properties as well as its crystallization behaviour. A batch of glass with the chemical composition of the residual glass, analysed using STEM-EDXS in the crystallized parent glass, was melted and analysed by methods including X-ray diffraction (XRD), electron backscatter diffraction (EBSD), XANES and multiple TEM techniques.

## Methods

The glass A with the composition stated in Table 1 was prepared from the raw materials SiO_2_, 4 MgCO_3_ · Mg(OH)_2_ · 5 H_2_O, Al(OH)_3_ and Y_2_O_3_. Batches of 150 to 250 g of this glass were melted at 1590 °C in a platinum crucible. The temperature was kept for 2 h before the melt was cast into water, dried and crushed to pieces with sizes ≤1.25 mm. In order to improve the homogeneity, the glass was re-melted at 1590 °C for another 2 h, and cast into a steal mould preheated to 600 °C. Subsequently, the glass was transferred to a cooling furnace preheated to 850 °C. The furnace was switched off to slowly cool the glass to RT with an approximate rate of 2 K min^−1^. The prepared glass A was cut into pieces and then crystallized by one- or two-step thermal treatments with heating and cooling rates of 5 K min^−1^. The samples were cooled to RT between the annealing steps. The annealing regimes of the resulting glass-ceramics A1–A7 are stated in Table 2. The glass-ceramics A3, A4 and A5 were prepared by applying the stated regimes to samples of the glass-ceramic A2. Samples of glass-ceramic A2 were powdered to a grain size of ca. 10 μm using a ball mill before the further heat treatment to obtain the glass-ceramics A4 and A5.

A residual glass was observed in the glass-ceramic A2 and its composition was calculated from STEM and EDXS analyses. The composition of this glass B is also stated in Table 1. It was melted with the same raw materials and by the same methods as glass A. Glass B was crystallized comparably to the glass-ceramics A1 and A2, resulting in the glass-ceramics B1 and B2. The glass-ceramic B3 was produced by applying the annealing regime stated in Table 2. As for glass A, all samples were cooled to RT between the annealing steps.

Differential Scanning Calorimetry (DSC, Linseis DSC Pt1600) was performed using grain sizes from 250 to 315 μm and supplying a heating rate of 5 K min^−1^. Dilatometric measurements (NETZSCH Dil 402 PC) were performed on cylindrical glass specimens with a diameter of 8 mm and a length of 25 mm using the same heating rate of 5 K min^−1^. A helium pycnometer (Accupyc 1330) was used for the measurement of the density at room temperature. The phase composition of powdered and bulk samples was determined using XRD (Siemens D5000 diffractometer) with CuK_α_ radiation (λ = 0.154 nm) in a 2θ range from 10° to 60°. The microstructure was studied using a scanning electron microscope (SEM Jeol JSM 7001F) equipped with an EDAX Trident analyzing system containing a Digiview 3 EBSD-camera. EBSD-scans were performed using a voltage of 20 kV and a current of ca. 2.40 nA. The scans were captured and evaluated using the software TSL OIM Data Collection 5.31 and TSL OIM Analysis 6.2. Unreliable data points were removed in all datasets used for orientation analyses by applying a Confidence Index (CI) filter of 0.1 after performing a grain CI standardization. No further cleanups which actually modify orientations were applied.

STEM analysis of the glass-ceramics was performed on an FEI Titan^3^ 80–300 electron microscope using an acceleration voltage of 300 kV with a high-angle annular dark field detector (HAADF, Fischione Model 3000). Energy dispersive X-Ray analyses (EDXS) were performed using a Super-X EDX detector equipped with four SDD detectors (FEI) to obtain element distribution mappings with the software *Esprit* (Bruker). Element mappings were derived by evaluating the lateral distribution of the peak intensity, i.e. the area underlying the *K*_*α*_ edges of the analyzed elements, with an automatic routine provided by the software. The STEM sample preparation was done by a purely mechanical wedge-polishing routine (polishing system Multiprep^TM^, Allied company), followed by a low-energy (2.5 keV) Ar^+^ broad beam final milling step (Gatan precision ion polishing system PIPS) to achieve electron transparency as well as to remove any residues from the mechanical polishing.

XANES measurements of the Zr *L*_*2*_- and Zr *L*_*3*_-edges were performed at the Canadian Light Source (CLS) in Saskatoon at the Soft X-ray Micro Characterization Beamline (SXRMB). Si(111) crystals were used as monochromators resulting in an energy resolving power of 10^4^. The samples were mounted on a sample holder using double-sided conducting carbon tape. The pressure in the vacuum chamber during the analyses was approximately 10^−8^ mbar. The fluorescence yield (FY) data were recorded using a Si-Li drift detector. The energy range between ca. 2,280 eV and 2,340 eV was scanned for the Zr *L*_*2*_-edge, while the energy range between ca. 2,210 eV and 2,240 eV was investigated for the Zr *L*_*3*_-edge. The FY data were normalized to the incident beam intensity (I_0_). Background subtraction was done with the commercially available software UNIFIT 2014^28^, where an atan((*E*-*E*_*s*_)/*β*_*s*_) approach is used to describe the background at the edge jump with the photon energy *E*, the edge position *E*_*s*_ and the full-width-half max (FWHM) of the step 2*β*_*s*_.

The Vickers’ hardness (microhardness) of the glasses and glass-ceramics was measured with a load of 1.96 N using a microhardness tester (Duramin 1, Struers) on samples with one polished side and a size of 5 × 5 × 10 mm^3^. 10 measurements were performed on each sample and the microhardness was calculated using the following equation (1)^29^:


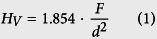


where *H*_*V*_ is the Vickers hardness in GPa, *F* is the supplied force in N and *d* is the mean length value of the indentation diagonals in m. The 4-point bending strength of 7 to 12 specimens with a size of 3 × 4 × 45 mm^3^ was measured using a universal testing machine (Zwick 1445) and a cross head speed of 1 mm min^−1^. Young’s moduli of 4 to 10 samples with a size of 3 × 4 × 60 mm^3^ were determined with the same testing machine (Zwick 1445) in a 3-point method. For both measurements, the samples were cut and polished before crystallization. The presented mechanical properties are mean values from which standard deviations were calculated.

## Results and Discussion

### Crystallization of the parent Glass A

**Figure 1:**
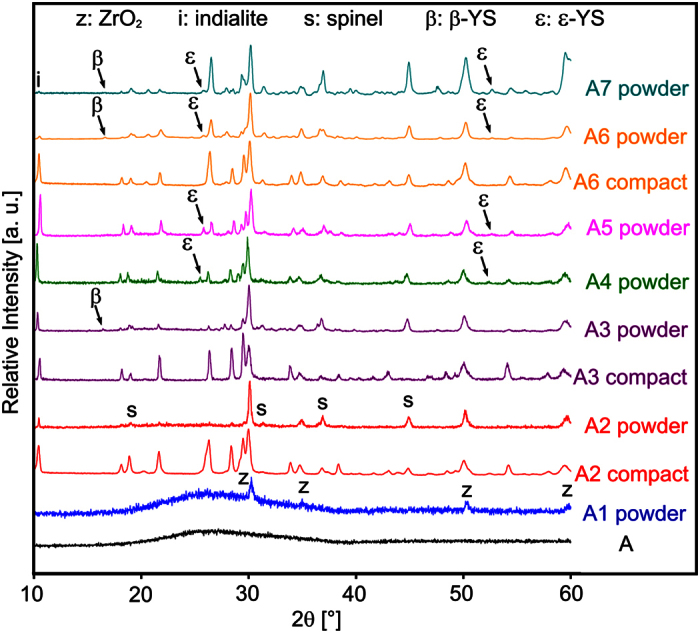
XRD-patterns of glass A and the glass-ceramics A1 to A7 crystallized from it according to the regimes stated in Table 2.

Figure 1 presents XRD-patterns obtained from the prepared glass A and the glass-ceramics A1 to A7 produced according to the tr**e**atments outlined in Table 2. The glass-ceramics A2, A3 and A6 were analyzed as compact samples and as powders. While the patterns obtained from powdered samples provide information on the bulk crystallization, those obtained from compact samples provide information concerning a layer of ≈100 μm below the sample surface, i.e. a possible surface crystallization, due to the finite penetration depth of the X-rays.

The XRD-pattern of glass A shows that it is X-ray amorphous, while three peaks of marginal intensity at 2θ ≈ 30°, 51° and 60° in the pattern of glass-ceramic A1 can be attributed to either cubic (JCPDS 07–0337) or tetragonal ZrO_2_ (JCPDS 50–1089). This also indicates that ZrO_2_ is the first phase to crystallize in glass A. Hence it is justified to assume that the corresponding XRD-peaks occur in the patterns of all glass-ceramics produced from glass A. The patterns of the compact and powdered glass-ceramic A2 both clearly indicate the presence of ZrO_2_ and indialite (Mg_2_Al_4_Si_5_O_18,_ JCPDS 75–1439) due to the very characteristic peak at 2θ = 10.2°. Although all peak positions of spinel (MgAl_2_O_4,_ JCPDS 21–1152) are superimposed by other phases possible in the system, i.e. the Y_2_Si_2_O_7_ (YS) phases β- and ε-YS, it is justified to state that spinel is indicated in both patterns because other peaks of the superimposing phases are not observed. However, the intensity of the peaks attributed to indialite is clearly reduced in the pattern of the powdered sample, indicating a preferred surface crystallization of indialite at the air-glass interface.

Samples of the glass-ceramic A2 were processed further using the schemes A3 to A5 in Table 2 to investigate whether the residual glass detected in A2 continues to crystallize. The relative intensities of the peaks attributed to spinel in the pattern of the compact glass-ceramic A3 are significantly reduced which may be explained by a thicker layer of indialite at the surface after longer crystallization. The XRD-pattern obtained from the powdered glass-ceramic A3 shows additional peaks attributable to β-Y_2_Si_2_O_7_ (β-YS, monoclinic, JCPDS 38–0440), especially the peak at 2θ = 16.5°. If the glass-ceramic A2 is powdered before the further processing, leading to the glass-ceramics A4 and A5, the XRD-patterns of the resulting glass-ceramic powders are basically identical and show the additional presence of ε-YS instead of the β-YS observed when crystallizing the compact sample (A3). The phase ε-YS (JCPDS 74–1994) is indicated by characteristic peaks at 2θ = 25.6° and ≈52.5°. This allows the conclusion that ε-YS preferably crystallizes at the interface to air. Glass-ceramic A6 was produced to analyse the continued crystallization of the amorphous matrix at 1060 °C when extending the second annealing step. The XRD-patterns of the compact and powdered samples are basically identical to those obtained from glass-ceramic A2 with the exception that the pattern of the powdered sample additionally indicates β-YS and maybe ε-YS. Glass-ceramic A7 was directly heated to 1060 °C without the first annealing step at 950 °C and the XRD-pattern of a powdered sample is basically identical to that of the powdered glass-ceramic A6 except for differences in the relative peak intensities.

**Figure 2:**
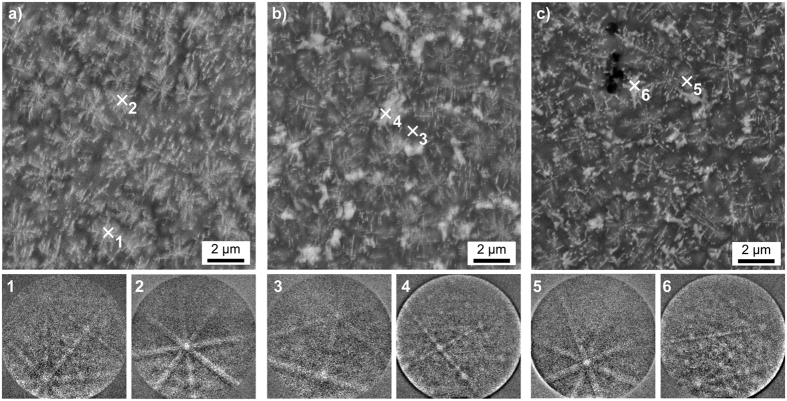
SEM micrographs of the bulk microstructure (**a**) in glass-ceramic A2, (**b**) in glass-ceramic A3 and (**c**) in glass-ceramic A6. The EBSD-patterns 1–6 were obtained at the locations marked by the crosses 1–6 in the respective samples.

The microstructure in the bulk of glass-ceramic A2 is presented in Fig. 2(a). The EBSD-patterns 1 and 2 were obtained from this microstructure at the locations 1 and 2. Pattern 1 may be reliably indexed as cubic ZrO_2_, but an exclusion of spinel cannot be achieved by the band position analysis alone because both phases have the same space group. However, applying the band width ratio matching leads to a much better d-space fit for ZrO_2_ where the a-axis is only approximately half as long as in spinel. The EBSD-pattern 2 could not be indexed reliably. Annealing this glass-ceramic again to produce the glass-ceramic A3 leads to the microstructure presented in Fig. 2(b), while extending the second annealing step to 20 h leads to the microstructure of glass-ceramic A6 shown in Fig. 2(c), where pores (dark) are observed. The EBSD-patterns 3–6 were obtained at the respectively marked locations. The patterns 3 and 5 basically match the characteristics of pattern 2, the patterns 4 and 6 originate from the large, bright structures which were not observed in Fig. 2(a). Pattern 4 may be reliably indexed as α-YS while pattern 6 may be reliably indexed as β-YS. By contrast, the XRD measurements in Fig. 1 only indicated the presence of β-YS in both glass-ceramics. Reliably detecting α-YS in this system by XRD is basically impossible because all major peaks of α-YS are superimposed by peaks of either indialite, spinel and/or β-YS which are all proven to occur in these glass-ceramics. Simultaneously, not detecting β-YS in glass-ceramic A3 by EBSD-spot measurements does not mean it cannot occur in the sample as indicated by XRD. Ultimately it is likely that both α- and β-YS occur in the glass-ceramics A3 and A6. EBSD-patterns indicating other YS, especially ε-YS detected in the glass-ceramics A4 and A5, could not be obtained from the bulk of the respective compact glass-ceramics. This is in agreement with the assumption that this phase preferably crystallizes at the interface of the residual glass with air. EBSD-patterns of ZrO_2_ were obtained from all analysed glass-ceramics. The EBSD-patterns 2, 3 and 5 are very similar to the unindexed patterns observed at the glass/ceramic-interface in ref. 10. The main difference between these patterns and the patterns indexable as ZrO_2_ is that they only contain one or two zone axes while the patterns reliably indexable as ZrO_2_ contain at least three. The few bands in these patterns from a probably cubic phase lead to an insufficient number of votes for a reliable indexing process. Considering that none of the EBSD-patterns analysed in these glass-ceramics are actually high quality patterns when compared to other systems, it seems quite possible that they originate from ZrO_2_ instead of an unknown phase and simply contain too few details to enable a reliable evaluation.

**Figure 3:**
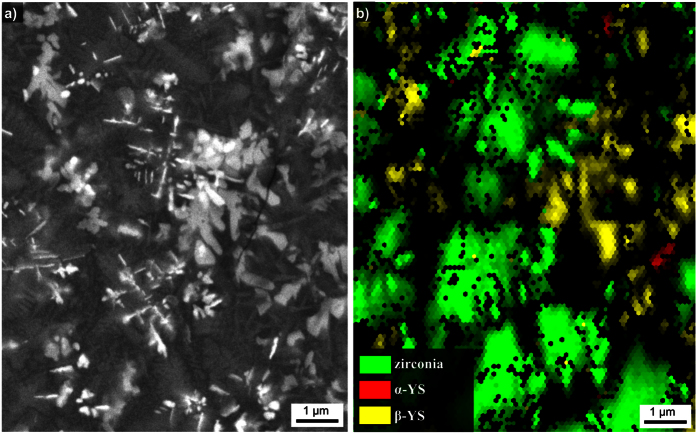
(**a**) SEM micrograph of the bulk microstructure of the glass-ceramic A7 after annealing the glass at 1060 °C for 20 h. (**b**) Phase-map of an EBSD-scan performed on a comparable area.

If the nucleation step is not included and glass A is directly heated to 1060 °C for 20 h, i.e. A7, the crystal growth occurs in slightly coarser, dendritic structures as shown in Fig. 3(a). Also, this microstructure contains cracks rather than the pores observed in Fig. 2(c). This microstructure allows the acquisition of significantly better EBSD-patterns, probably due to the larger crystals. The phase map of an EBSD-scan performed with a step size of 100 nm is presented in Fig. 3(b) to show that the ZrO_2_ and β-YS dominantly occur while small pockets of α-YS are also observed. This is in agreement with the XRD pattern of this glass-ceramic in Fig. 1 considering that the peaks of α-YS are again superimposed by the peaks of indialite, spinel and/or the β-YS and ε-YS probably only occurs near the surface.

**Figure 4:**
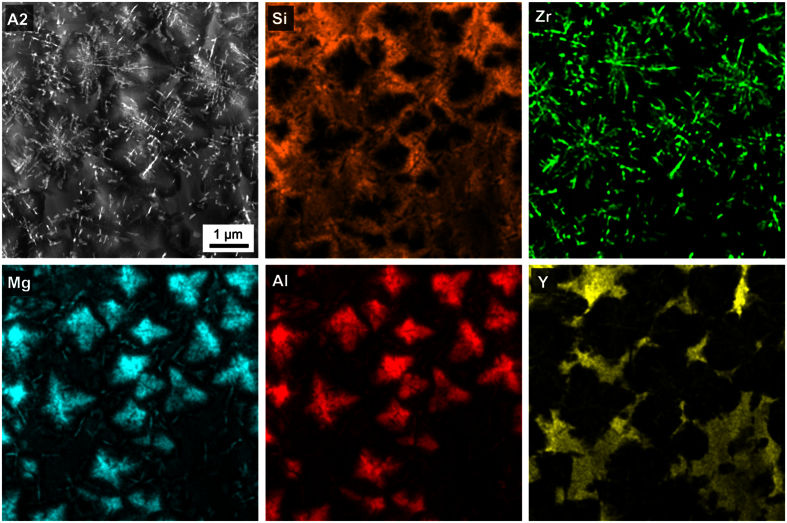
STEM-HAADF micrograph and respective element mappings of Si, Mg, Al, Zr and Y of glass-ceramic A2 crystallized at 950 °C for 5 h and at 1060 °C for 1 h.

Figure 4 shows an STEM-HAADF micrograph of the glass-ceramic A2. Clearly, a phase of dendritic appearance is observed, as well as areas of blurry, less bright contrast. HAADF imaging is based on the inelastic electron scattering of the primary electron beam in interaction with the atoms of the sample. Hence the resulting contrast and thus the brightness of the micrograph are very sensitive to the mass of the occurring atoms. The scattering cross section depends on the atomic number *z* with approximately *z*^*a*^, where *a* varies between 1.2 and 1.8 for the used type of TEM and HAADF detector^30^. The heaviest elements in the sample are Zr and Y, making STEM-HAADF imaging well-suited to locate Zr- and Y- rich areas. Both XRD and EBSD show that crystalline ZrO_2_ should occur in the glass-ceramic A2. The morphology of the Zr enrichment indicated in the corresponding element distribution in Fig. 4 enables to conclude that the dendritic structures in Fig. 4 consist of ZrO_2_ which is in agreement with the EBSD-patterns acquired from similar structures in Fig. 2. The large, rather homogeneous areas of blurry, bright contrast in the STEM-HAADF micrograph are enriched in Y. The comparably large, blocky structures enriched in Mg and Al, while depleted of Zr, Y and Si should be the spinel (MgAl_2_O_4_) as indicated by XRD in Fig. 1. As no further crystalline phases are indicated in the bulk of sample A2 and EBSD-patterns could not be obtained from the dark grey phase in Fig. 2(a), the phase of blurry, bright contrast between the crystals is most likely the residual glass matrix. It is enriched in Si and especially in Y, but depleted of Mg, Al and Zr^6^. The quantitative analysis of this residual glass with EDXS resulted in the mol% composition 54.7 SiO_2_ · 10.9 Al_2_O_3_ · 15.0 MgO · 3.4 ZrO_2_ · 16.0 Y_2_O_3_^6^.

### Crystallization of the residual glass B

To further analyze this residual glassy phase and its properties free of any crystalline components, a glass of the same composition was melted and denoted as glass B. Glass B was studied by SEM and TEM (not shown here) without detecting any heterogeneities. Quantitative STEM-EDXS was applied to glass B to ensure its comparability to the residual glass in the glass-ceramic A2. The determined mol% composition of glass B is 54.1 SiO_2_ · 11.1 Al_2_O_3_ · 14.5 MgO · 3.8 ZrO_2_ · 16.3 Y_2_O_3_, meaning both glasses have the same composition within the margin of error.

**Figure 5:**
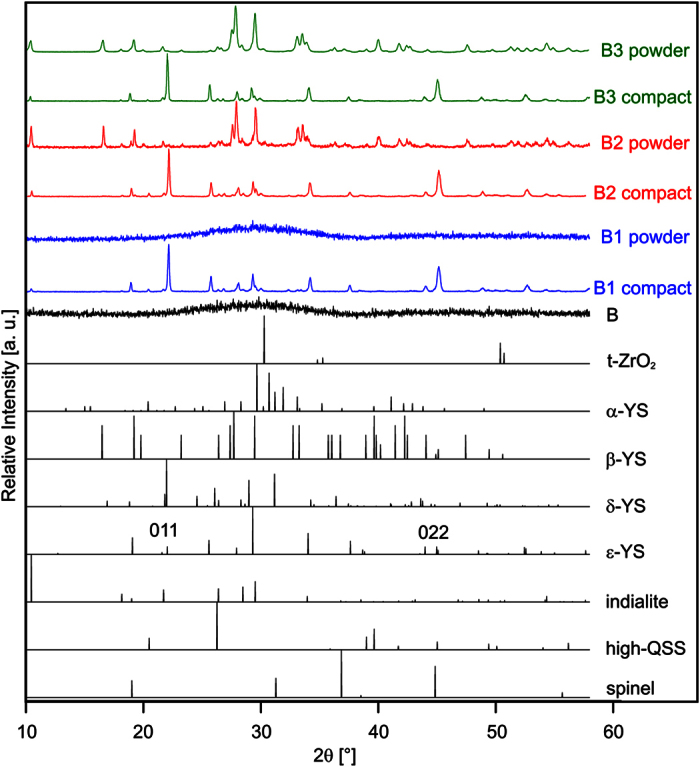
XRD-patterns of glass B and the glass-ceramics crystallized from it according to the regimes stated in Table 2. Theoretical patterns of possible crystal phases are presented below for comparison and to outline the problem of peak superposition.

The crystallization behavior of the melted residual glass B in sole contact to air was studied by annealing it according to the regimes B1 to B3 stated in Table 2. Figure 5 presents XRD-patterns obtained from glass B as well as from compact samples and powders of the glass-ceramics B1 to B3. The theoretical patterns of some YS-phases as well as of tetragonal ZrO_2_, spinel, indialite and the high-QSS are presented below for comparison and to visualize the problem of peak superposition already outlined above in the context of Fig. 1. While the glass B is X-ray amorphous, the patterns of the compact glass-ceramics B1 to B3 indicate the presence of multiple crystalline phases with many peak superpositions, making a correct phase attribution difficult.

The patterns of the compact samples basically show the same result. Peaks not superimposed by other possible phases indicate the presence of indialite (2θ = 10.2°) and ε-YS (2θ = 25.6° and ≈52.5°). The peaks of comparably high intensity at ca. 22.1° and 45.0° could indicate a preferred orientation of ε-YS with its 0nn-planes parallel to the surface, but they could be superimposed by peaks of α- and/or δ-YS (orthorhombic, JCPDS 76–0204, 100% peak at 2θ = 22.0°), thus making an evaluation of the peak intensities unreliable. The pattern of the powdered glass-ceramic B1 shows no discernible peaks. Considering the pattern obtained from the compact glass-ceramic B1, this indicates that a sole surface crystallization is observed and the crystallized layer in the compact sample did not contain enough volume to be detected after powdering. The XRD-patterns of the powdered glass-ceramics B2 and B3 are almost identical and show very different peaks compared to those of the compact glass-ceramics. They indicate the presence of indialite, β-YS (2θ = 16.5° and 47.5°) and only traces of ε-YS. Altogether these XRD patterns indicate a preferred crystallization of ε-YS near the surface while the bulk mainly contains β-YS. This is in agreement with the observation that ε-YS preferably crystallizes at the air/glass interface, i.e. the surface, as indicated by the glass-ceramics A4 and A5 in Fig. 1.

**Figure 6:**
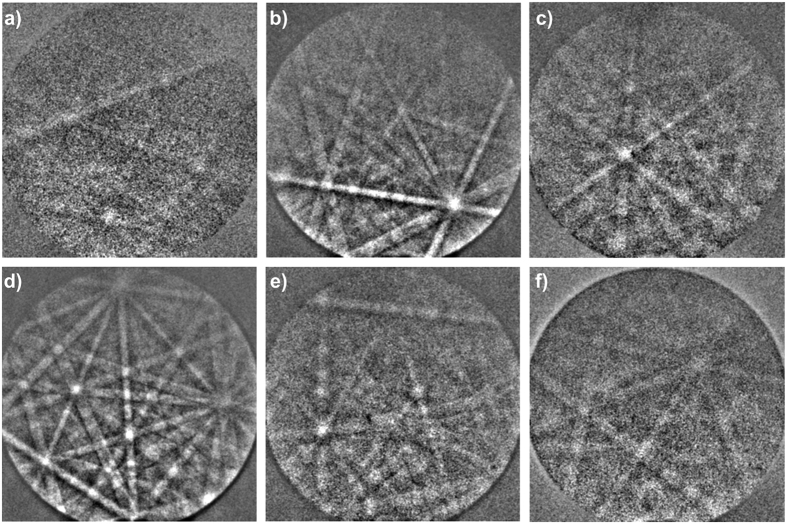
EBSD-patterns of different phases acquired from glass-ceramics of glass B. Selected indexing parameters are stated in Table 3.

The presence of peaks attributable to the β-, δ- and ε-YS in the XRD-patterns of Fig. 5 calls for a more detailed phase analysis because at least six different yttrium silicates of the composition Y_2_Si_2_O_7_ are currently known^31^. The XRD-analysis of these phases in the given θ-2θ-setup has led to incorrect phase identifications in the past which could be corrected by local EBSD analyses^32^. Figure 6 presents EBSD-patterns acquired from glass-ceramics of glass B, annealed for 5 h at 950 °C and another 1 h at 1060 °C (glass-ceramic B2). The selected indexing parameters presented in Table 3 show that indialite, α-, β-, δ-, and ε-YS as well as a cubic phase of unknown composition, probably ZrO_2_, were located after crystallization. However, peaks clearly attributable to α-YS could not be observed in the XRD-patterns due to peak superposition. A detailed description of the multiphase microstructure observed in this glass-ceramic is beyond the scope of this article. It must be noted that the sole surface crystallization observed in many glasses of the MAS system is also observed here in agreement with the XRD-results of glass-ceramic B1 in Fig. 5. Bulk nucleation does not occur.

**Figure 7:**
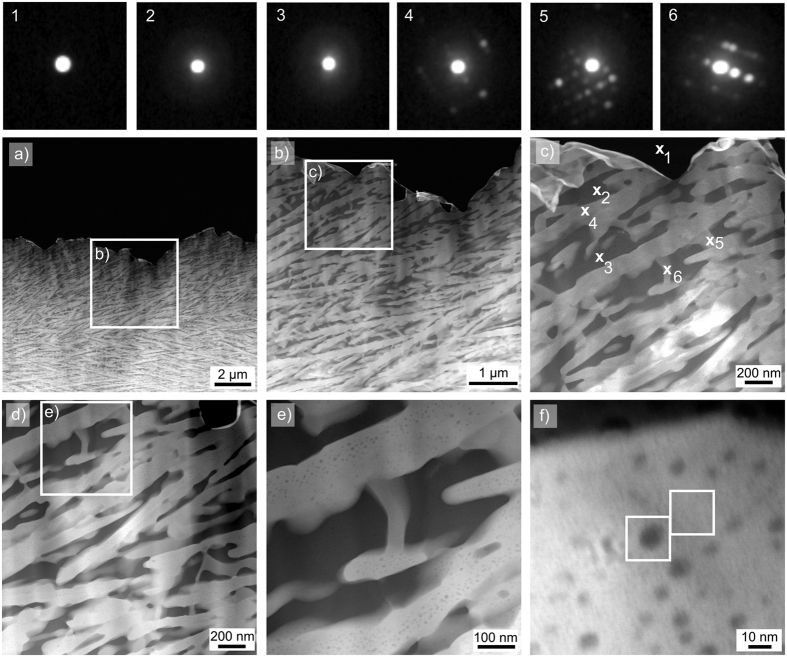
STEM micrographs of a sample of glass-ceramic B3. The nano-diffraction patterns 1–6 were obtained at the locations 1–6 in (**c**). The area presented in (**e**) and the framed areas in (**f**) are further analyzed in Figs 8 and 9.

**Figure 8:**
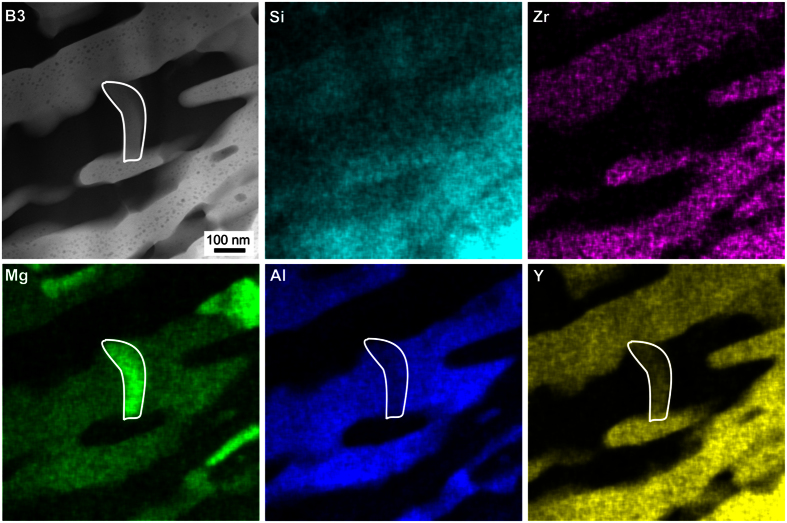
STEM-HAADF micrograph of an area in glass-ceramic B3 denoted as (e) in Fig. 7 and respective element mappings of Si, Zr, Mg, Al and Y.

Figures 7 and 8 show STEM-HAADF micrographs and EDXS mappings recorded from the bulk of glass-ceramic B3. The STEM micrographs in Fig. 7(a–c) show the existence of dendritic structures of bright contrast which grew in a matrix appearing dark in the STEM-HAADF images. Qualitative nano-diffraction experiments were performed at several spots of this microstructure as shown in Fig. 7(c) to test this matrix for crystallinity. The spot-like diffraction pattern 1 was taken in vacuum. The patterns 2 and 3 of the dark areas show ring-like features, confirming them to be amorphous. The diffraction patterns 4 to 6, however, show distinct diffraction spots for measurement positions on the bright dendrites, confirming their crystallinity. Hence, the dark areas constitute the residual glass in the glass-ceramics of glass B, which in turn was the residual glass of glass-ceramic A2. The STEM micrographs with higher magnifications in Fig. 7(e,f) show that the bright phase is not homogeneous itself, but includes circular areas of a darker contrast which indicates the presence of a higher concentration of lighter elements in these areas, e.g. Mg or Al.

Figure 8 presents the STEM-EDXS element maps of Al, Si, Zr, Mg and Y of the area shown in Fig. 7(e). The bright phase in the HAADF-micrograph is enriched in Si, Y and Zr, while Al and Mg are not observed in these areas, although they probably occur in the dark inclusions visible in Fig. 7(f) but not resolved with the chosen magnification of Fig. 8. Apparently, the bright, crystalline phase is one of the YS-phases detected by EBSD and XRD. It is probably the β-YS predominantly found in the bulk of the glass-ceramics. An EDXS-based quantification of the element composition of this crystalline phase resulted in an approximated element ratio of Mg:Al:Si:Zr:Y ≈ 4:5:45:7:40, but it must be noted that the dark, spherical structures with a diameter of up to 10 nm observed in Fig. 7(f) also contribute to the EDXS signal.

**Figure 9:**
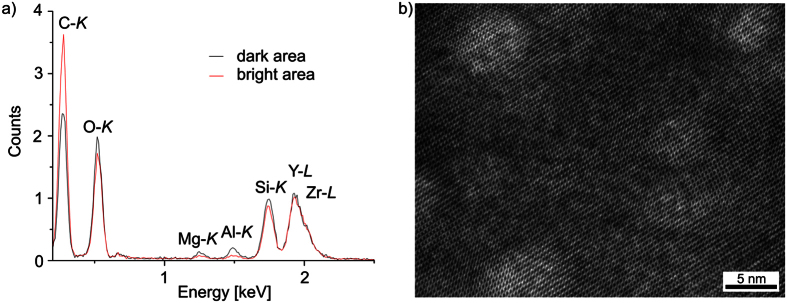
(**a**) EDX-spectra of the framed areas in Fig. 7(f) and (b) HR-TEM micrograph of an area of glass-ceramic B3, comparable to an area as shown in the STEM-HAADF micrograph of Fig. 7(f).

Figure 9(a) presents the EDX spectra of the two sub-areas framed in Fig. 7(f). Comparing them shows that Mg and Al are enriched in the dark areas of the micrograph. The crystal lattice of such a dark area is visualized by the high-resolution TEM (HR-TEM) micrograph shown in Fig. 9(b). Apparently, the crystal lattice of the YS-phase does not show any recognizable distortion at the positions of the spherical inclusions, which may indicate that these structures are amorphous but some layers of the YS are either above or below the inclusion in the prepared TEM sample. As they are enriched in Mg and Al (see Fig. 9(a)), it is likely that the growing YS expels Mg as well as Al and surrounds such areas to form these spherical inclusions.

Using STEM-EDXS, another phase with an element ratio of Mg:Al:Si:Zr:Y ≈ 30:22:30:2:15 was detected in the framed area of Fig. 8. Here, the concentrations of Mg and Y are increased, while Zr is depleted. The crystallinity of this phase is proven by the nano-diffraction pattern in Fig. 7(c), but it is not attributable to any of the crystal phases, which were detected in the XRD-patterns in Fig. 5 and in the EBSD analyses in Fig. 6. It seems likely that this structure is also a YS-phase but covered by a thin glassy layer in the prepared TEM-sample which would explain the deviating composition of more Si, Mg and Al. It is worth noting that the composition of the residual glass in the glass-ceramic B3 is close to the composition of the original glass A: glass A shows the element ratio of Mg:Al:Si:Zr:Y ≈ 17:34:41:4:4 while the residual glass of glass-ceramic B3 has an element ratio of Mg:Al:Si:Zr:Y ≈ 18:39:40:2:2.

The results outlined so far indicate some clear differences between the crystallization of glass A and glass B. While glass A shows surface and bulk nucleation, a sole surface nucleation is indicated in glass B. ZrO_2_ is observed in both glass-ceramics, but while it serves as a nucleation agent in glass A, this cannot be said for glass B due to the lack of bulk nucleation. In addition to ZrO_2_, indialite and α-, β- as well as ε-YS are detected in glass-ceramics produced from both glasses, spinel is only proven in glass A while δ-YS is only proven in glass B. Indialite as well as ε-YS show a preferred crystallization at the surface while α- and β-YS are predominantly observed in the bulk. The high- or low-QSS are not observed in any glass-ceramic with certainty due to the problem of peak superposition in the XRD-patterns. However, it should be noted that these phases are often observed in related glass-ceramics^4^,^5^,^6^,^7^,^8^,^9^,^10^ and the chemical circumstances for their formation are given in this system.

### Coordination of Zr in glass B

**Figure 10:**
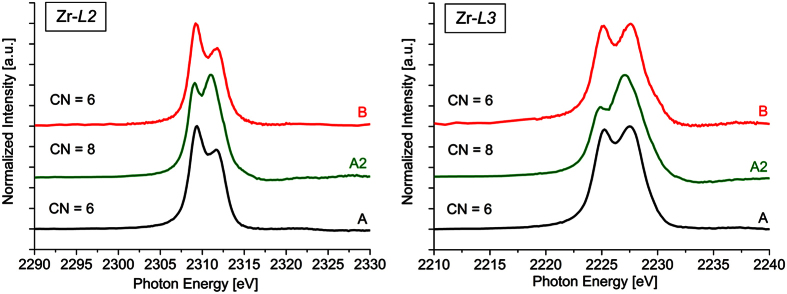
XANES spectra of the Zr *L*_*2*_- and Zr *L*_*3*_- edges of the glasses A and B and the glass-ceramic A2.

XANES spectra recorded at the Zr *L*_*2*_- and Zr *L*_*3*_- edges from the glasses A and B as well as from the glass-ceramic A2 are presented in Fig. 10. Two features at ≈2,309 and 2,312 eV are observed in the spectra of the Zr *L*_*2*_- edge while the spectra at the Zr *L*_*3*_- edge show two features at ≈2,225 and 2,227 eV which correspond to 2p → 4d electron transitions. Both edges in the spectra of the glasses A and B indicate a 6-fold coordination of Zr in agreement with previous results on MAS glasses with similar chemical compositions^33^. By contrast, the spectra of the glass-ceramic A2 show different peak forms at both edges, representing an increased coordination number of Zr^4+^ 33,34. The crystallization of ZrO_2_ in glass A is thus accompanied by a change in the coordination number: while Zr^4+^ is incorporated into the glass network in a 6-fold coordination, it is 8-fold coordinated in the crystallized, tetragonal (or cubic) ZrO_2_^33^. However, the transition from the 6- to the 8-fold coordination is not completed in the glass-ceramic A2, because a certain amount of Zr remains in the residual glass after crystallization^6^,^23^. In previous investigations, it was assumed that the Zr in the residual glass still exhibits the 6-fold coordination^23^. However, a direct experimental proof was not presented yet because the lateral expansions of the residual glass areas in the glass-ceramic A2 (≈1 μm or less) are too small for a direct analysis by XANES due to the limited lateral resolution of this technique. Now this assumption is underlined by the XANES measurement of the re-melted glass B, which undoubtedly shows a 6-fold coordination of this residual glass.

### Thermal and mechanical properties of the glasses A and B

Table 4 presents the CTE, *T*_*g*_ and the density of the parent glass A and the residual glass B. Glass B exhibits a much higher *T*_*g*_ than the parent glass A (840 °C vs. 818 °C), a higher density (3.54 g/cm^3^ vs. 2.88 g/cm^3^) and a larger CTE (6.5 · 10^−6^ K^−1^ vs. 5.5 · 10^−6^ K^−1^) in a range from 100 to 500 °C. The increased density of glass B can be explained by the higher concentration of heavy Y atoms compared to glass A. The increase in the CTE and in *T*_*g*_ due to the addition of Y_2_O_3_ has previously been reported from a BaO-CaO-Al_2_O_3_-SiO_2_ glass^35^. This is in agreement with the higher CTE and *T*_*g*_ of glass B, which contains a much higher concentration of Y_2_O_3_ than the original glass A.

Table 4 also presents the mechanical properties of the glasses A and B, as well as those of the glass-ceramics A2. The mechanical properties of glass-ceramic B2 could not be measured due to its high fragility. Holes can be observed at the fractured areas of these samples, which may be responsible for their high fragility. Glass B shows exclusive surface crystallization of multiple phases, resulting in a higher density of the outer crystallized layer. During crystallization, material from the bulk diffuses to the crystallization front, which may result in cracks and holes in the bulk. By contrast, the glass-ceramic A2 shows the volume crystallization of phases with large CTEs. Spinel has a CTE of α_20–800 °C_ = 8 · 10^−6^ K^−1^ 22 and tetragonal ZrO_2_ of α = 10.5 · 10^−6^ K^−1^ 24, while the residual glass B has a CTE of α = 6.5 · 10^−6^ K^−1^. Due to the mismatch between theses CTEs, the glass-ceramic A2 exhibits a high bending strength of σ = 356 MPa, while glass A only shows a bending strength of σ = 98 MPa. The residual glass in glass-ceramic A2, *i.e*. glass B, nearly has the same bending strength of σ = 104 MPa. Furthermore, the microhardness of the glasses A and B is equal for both compositions with H_V_ = 8.4 GPa and increases after thermal treatment. The glass-ceramic A2 exhibits a microhardness of H_V_ = 9.9 GPa. The Young’s moduli for the glasses A and B as well as for sample A2 are between E = 102–114 GPa and not significant different, considering the limits of error.

## Conclusions

An yttria-containing glass in the MAS-system (glass A) was crystallized in two steps at 950 °C for 5 h and at 1060 °C for 1 h. This resulted in the bulk crystallization of ZrO_2_ and spinel, and the surface crystallization of indialite, all embedded within a residual glass matrix. The chemical composition of the latter was calculated based on STEM-EDXS analyses. In order to further analyse this residual glass, a batch of glass with this composition (glass B) was melted. Its CTE, *T*_*g*_ and density values are higher than the respective values of the original parent glass A. The CTE of 6.5 · 10^−6^ K^−1^ may explain the previously reported high mechanical properties of the glass-ceramic A2, due to the mismatch between this CTE value and those of the crystal phases spinel (CTE = 8 · 10^−6^ K^−1^ 22) and ZrO_2_ (CTE = 10.5 · 10^−6^ K^−1^ 24). The mechanical properties of the residual glass and the original glass are similar. The coordination of Zr in the residual glass was determined by XANES measurements to be 6-fold, as in the parent glass.

The crystallization of the residual glass within the glass-ceramic A2, reached by further annealing, was compared with the crystallization of glass B. As glass B is only in contact with air during annealing but the residual glass in glass-ceramic A2 is in the contact with multiple crystalline phases, some differences are observed. Treating the glass-ceramic A2 with further annealing steps leads to the growth of α- and β- YS. The phase ε-YS is additionally detected if the air/glass-interface of the glass-ceramic A2 is significantly increased by powdering it before annealing. Glass B shows a strong surface crystallization of indialite and ε-YS, while α-, β- and δ-YS as well as ZrO_2_ are also detected. Spinel and the high- or low-QSS could not be reliably detected in the glass-ceramics produced from glass B.

## Additional Information

**How to cite this article**: Seidel, S. *et al*. Characterizing the residual glass in a MgO/Al_2_O_3_/SiO_2_/ZrO_2_/Y_2_O_3_ glass-ceramic. *Sci. Rep*. **6**, 34965; doi: 10.1038/srep34965 (2016).

## Figures and Tables

**Table 1:** Compositions of the melted glass A and the residual glass B after the two-step crystallization at 950 °C for 5 h and at 1060 °C for 1 h (A2).

	Chemical composition in mol%				
	SiO_2_	Al_2_O_3_	MgO	ZrO_2_	Y_2_O_3_
glass A	50.6	20.7	20.7	5.6	2.4
glass B	54.7	10.9	15.0	3.4	16.0

**Table 2:** Thermal treatment of the glass-ceramics.

Glass-ceramics	Preparation/thermal treatment
A1 and B1	950 °C for 5 h
A2 and B2	950 °C for 5 h + 1060 °C for 1 h
A3	**A2** + 950 °C for 5 h + 1060 °C for 1 h
B3	950 °C for 5 h + 1060 °C for 5 h
A4	**A2** → powdered → 950 °C for 5 h
A5	**A2** → powdered → 950 °C for 5 h + 1060 °C for 1 h
A6	950 °C for 5 h + 1060 °C for 20 h
A7	1060 °C for 20 h

**Table 3:** ICSD-file no., phase and selected indexing parameters for the EBSD-patterns presented in Fig. 6.

Pattern	ICSD file	Phase	Votes	Fit [°]	CI
a)	30947	indialite	35	1.07	0.123
b)	164148	α-Y_2_Si_2_O_7_	84	0.73	0.232
c)	281312	β-Y_2_Si_2_O_7_	147	0.62	0.577
d)	33721	δ-Y_2_Si_2_O_7_	83	0.94	0.214
e)	28004	ε-Y_2_Si_2_O_7_	187	0.49	0.164
f)	fcc cubic	?	72	0.96	0.286

**Table 4:** CTE_(100–500 °C)_, T_g_, density, Vickers hardness (microhardness) H_V_, Young’s modulus E and bending strength σ_BB_ of the glasses A and B.

Sample	CTE_(100–500 °C)_ [10^−6^ K^−1^]	T_g_ [°C]	Density [g/cm^3^]	H_V_ [GPa]	E [GPa]	σ_BB_ [MPa]
A	5.5	818	2.88	8.4 ± 0.1	107 ± 2	98 ± 15
A2				9.9 ± 0.1	102 ± 20	356 ± 37
B	6.5	840	3.54	8.4 ± 0.1	114 ± 2	104 ± 18

The mechanical properties of the glass-ceramic A2 are also presented. The values of A and A2 were taken from ref. 7.
